# Effects of Dietary Iron Levels on the Production Performance, Nutrient Digestibility, Blood Biochemistry, and Meat and Fur Quality of Growing Rex Rabbits

**DOI:** 10.3390/ani15020274

**Published:** 2025-01-19

**Authors:** Jiali Chen, Jiyuan Wang, Lei Liu, Fuchang Li

**Affiliations:** Key Laboratory of Efficient Utilization of Non-Grain Feed Resources (Co-Construction by Ministry and Province), Ministry of Agriculture and Rural Affairs, Shandong Provincial Key Laboratory of Animal Nutrition and Efficient Feeding, Department of Animal Science and Technology, Shandong Agricultural University, Tai’an 271017, China; jlchen@sdau.edu.cn (J.C.); wangjiyuan96224@163.com (J.W.)

**Keywords:** fur quality, growth performance, iron metabolism, meat quality, rabbit

## Abstract

The objective of this study was to explore the effects of dietary iron (Fe) levels on the production performance, nutrient digestibility, blood biochemistry, and meat and fur quality of growing Rex rabbits. Two hundred 3-month-old Rex rabbits were randomly allocated to five groups, each with forty replicates. Rabbits were fed a basal diet supplemented with varying levels of Fe (0, 20, 40, 80, and 160 mg/kg) in the form of ferrous sulfate monohydrate. The analyzed Fe concentrations in the diets were 8.2, 25.4, 49.1, 85.6, and 178.7 mg/kg, respectively. Over a 35-day trial period, rabbits supplemented with 40 mg/kg Fe showed a markedly increased average daily feed intake, average daily gain, and fur area compared to the control group (*p* < 0.05). The addition of 20 and 40 mg/kg Fe markedly improved the digestibility of crude protein and nitrogen (*p* < 0.05). Additionally, 80 mg/kg Fe supplementation significantly increased the redness of the *longissimus dorsi* muscle 30 min postmortem (*p* < 0.05). Dietary Fe addition also significantly elevated serum concentrations of Fe and copper (*p* < 0.05) while decreasing the total Fe-binding capacity (*p* < 0.05). In conclusion, dietary Fe supplementation boosted growth performance, protein and nitrogen digestibility, Fe stores, and meat and fur quality in Rex rabbits. Specifically, 40 mg/kg Fe (diet Fe content of 49.1 mg/kg) improved growth performance, nutrient digestibility, and fur quality, while 80 mg/kg Fe (diet Fe content at 85.6 mg/kg) was optimal for enhancing meat quality.

## Simple Summary

The trace element iron (Fe) is crucial for various physiological processes, including hemoglobin production, electron transfer, binding, and adenosine triphosphate (ATP) synthesis. The Rex rabbit, a domestic breed valued for both fur and meat production, has been understudied in terms of its specific Fe requirements. The current study was conducted to investigate the impacts of dietary Fe levels on the production performance, nutrient digestibility, and blood biochemistry of growing Rex rabbits, with the goal of providing theoretical guidance for optimizing Rex rabbit production. Our findings indicate that dietary Fe supplementation at 40 mg/kg (total diet Fe content of 49.1 mg/kg) improves growth performance, protein and nitrogen digestibility, and fur quality in growing Rex rabbits. Furthermore, the optimal Fe addition of 80 mg/kg (total diet Fe content of 85.6 mg/kg) was found to improve meat quality in these animals.

## 1. Introduction

As an essential trace element in rabbits, iron (Fe) plays a critical role in various physiological processes, including hemoglobin production, electron transfer, binding, and adenosine triphosphate production [1]. Insufficient Fe intake can lead to Fe deficiency in erythroblasts and Fe-deficiency anemia, resulting in growth retardation [2]. Conversely, excessive Fe accumulation can cause cytotoxicity, triggering oxidative stress and widespread tissue and organ damage, potentially leading to various diseases [3]. Therefore, maintaining an optimal Fe status is crucial for ensuring proper bodily functions.

In recent years, the demand for rabbit meat and fur has steadily increased in both domestic and international markets, driven by improvements in living standards. The Rex rabbit, renowned for its high-quality fur and meat, has become one of the most widely farmed domestic rabbit breeds worldwide. This breed is characterized by strong growth development and fur follicle differentiation capabilities, a high reproductive efficiency, and a short production cycle [4,5,6]. Despite the economic importance of Rex rabbits, there is limited literature available on the evaluation of their dietary Fe requirements. Liu et al. [7] conducted research on the Fe nutritional requirements of 60-to-90-day-old New Zealand rabbits and found that optimal Fe supplementation in the diet elevated the growth and slaughter performances and promoted the development of organs. A previous study on Chinese yellow broilers showed that dietary Fe addition did not affect growth performance, immune organ coefficients, or Fe stores, but improved the meat quality [8]. Rincker et al. [9] indicated that dietary Fe supplementation elevated the growth performance and Fe stores of pigs, and the reduction in Fe stores might lead to the occurrence of anemia during the grower and finisher periods. Given the significance of Fe in animal nutrition and the lack of comprehensive research on Fe supplementation in Rex rabbits, it is essential to determine the optimal level of Fe supplementation in their diets.

This study aimed to investigate the impacts of dietary addition with different levels of Fe on the production performance, nutrient digestibility, blood biochemistry, and meat and fur quality of Rex rabbits, elucidating the underlying mechanisms responsible for the positive effects of Fe addition in this breed. The findings contribute to a better understanding of the nutritional Fe requirements of Rex rabbits, potentially improving their health, productivity, and overall welfare. This research may also have broader implications for the rabbit farming industry by providing valuable insights into optimizing dietary Fe levels for enhanced production outcomes.

## 2. Materials and Methods

### 2.1. Animals and Treatments

Two hundred 3-month-old Rex rabbits (100 males and 100 females) with a similar body weight (BW; 1947 ± 211 g) were randomly divided into one of the following five groups: Fe0 (control), Fe20, Fe40, Fe80, and Fe160. Each group contained 40 replicates, with each rabbit considered as an independent replicate. The rabbits were fed a basal diet with the addition of 0, 20, 40, 80, and 160 mg/kg Fe in the form of ferrous sulfate monohydrate (FeSO_4_·H_2_O), resulting in Fe concentrations of 8.2, 25.4, 49.1, 85.6, and 178.7 mg/kg, respectively. Following a 7-day adaptation period, rabbits were fed the assigned diets for 35 days. The basal diet (Table 1) was formulated, as recommended by the NRC (1977) [10], to meet the nutrient requirements set for growing rabbits. Approximately 200 mg of the feed sample was collected using the quartering method and stored at −20 °C for the measurement of nutrient levels. The Fe concentration in the feed of each group was measured using the atomic absorption spectroscopy method [2]. All rabbits were kept in individual cages with unlimited access to water and feed. The housing temperature, relative humidity, and lighting were controlled to simulate commercial conditions (21 ± 2 °C, 50–80%, with a 12 h dark/12 h light cycle). The BW of each rabbit was measured individually on the morning of day 36 after overnight fasting. Weekly feed intake data were collected throughout the experiment to calculate the average daily gain (ADG), average daily feed intake (ADFI), and feed conversion ratio (FCR).

### 2.2. Sampling

On day 35 of the trial, eight rabbits from each treatment group, chosen to represent the average BW of the group, were randomly chosen for blood sampling and tissue collection. Blood samples (10 mL) were collected from the heart [11], with 2 mL placed in a routine blood tube for immediate hemoglobin (HB) measurement; the remaining blood was centrifugated (3000 rpm, 15 min) to harvest the serum and then stored in the refrigerator (−20 °C) for less than four weeks. Subsequently, the rabbits were slaughtered humanely following electric stunning, and the *longissimus dorsi* muscle was collected on the basis of the procedure described by Li et al. [12].

### 2.3. Determination of Organ Coefficient and Meat and Fur Quality

After the Rex rabbits were slaughtered, the spleens, livers, and kidneys were weighed, and the organ coefficients were computed by dividing the organ weight by the BW of the corresponding rabbits [13]. The pH value and color parameters [L* (lightness), a* (redness), and b* (yellowness) values] of the *longissimus dorsi* muscle at 30 min postmortem, as well as drip loss, were measured according to Li et al. [12]. Additionally, the skin was opened along the midline of the abdomen. The fur width (measured at the narrowest point of the abdomen) and length (from the neck to the base of the tail) were recorded using a flexible tape measure [14]. The fur area was calculated by multiplying its length and width. Fur thickness and hair length were measured using a caliper. Each measurement was performed three times, and the average value was used for the analysis.

### 2.4. Measurement of Serum Biochemical Parameters and Immunoglobulins

Biochemical parameters in the serum, including alanine aminotransferase (ALT), alkaline phosphatase (ALP), aspartate aminotransferase (AST), albumin (ALB), total protein (TP), serum urea (UREN), glucose (GLU), calcium, and phosphorus levels, were determined using reagent kits purchased from Fujifilm Wako Pure Chemical Corporation (Osaka, Japan), following the manufacturer’s instructions. Measurements were performed using a Hitachi 7020 automatic biochemical analyzer (Hitachi, Tokyo, Japan). The concentrations of immunoglobulin (Ig) G, IgM, and IgA were examined with the ELISA kits (Nanjing Jiancheng Bioengineering Institute, Nanjing, China) on the basis of the protocols [15].

### 2.5. Determination of Serum Iron Metabolism Indicators and Trace Elements

Serum HB was determined with an automatic hematological analyzer (Abacus Junior Vet, Vienna, Austria) [13]. The levels of serum succinate dehydrogenase (SDH), ferritin (Fn), and transferrin (Tf) were measured using ELISA kits, and the concentrations of total Fe-binding capacity (TIBC), Fe, zinc, and copper were determined using colorimetric assays [16]. All reagent kits were purchased from the Nanjing Jiancheng Bioengineering Institute. Serum manganese was quantified using flame atomic absorption spectrometry [17].

### 2.6. Chemical Analyses and Calculation

On day 29 of the trial, eight rabbits per group were chosen and moved into metabolism cages (60 cm × 40 cm × 40 cm) for a 7-day trial, including three days for adaptation to cages and four days for feces and urine collection. Fecal samples were collected into the bags, and 10% sulfuric acid was added at a ratio of 1:5 volume-to-weight. The urine samples were collected into a container containing 20 mL 10% sulfuric acid and stored at 4 °C subsequently. Additionally, the ADFI, fecal output, and urine volume were recorded. After the samples were pooled, homogenized, and heat-dried [18], dry matter (DM), crude fiber (CF), crude protein (CP), acid detergent fiber (ADF), neutral detergent fiber (NDF), lignin, crude fat, ash, calcium, phosphorus, Fe, manganese, and zinc contents in the feed and feces, as well as the CP content in the urine, were determined according to AOAC methods [19]. Nutrient digestibility was calculated using acid-insoluble ash as an indigestible marker, as described previously [18,20,21].

### 2.7. Statistical Analysis

Individual rabbits were used as the experimental unit for all analyses. As no interaction effects were observed between the sex and Fe level, data analysis was conducted using one-way ANOVA of SAS 9.4 (SAS Institute Inc., Cary, NC, USA) with the Fe level as the only main effect after the assessment of data normality by the Shapiro–Wilk test. Duncan’s multiple-range test was performed to compare the statistical differences among groups. The results are expressed as means with the RMSE. A level of *p* < 0.05 was regarded as statistically significant.

## 3. Results

### 3.1. Effects of Dietary Fe Levels on Growth Performance of Growing Rex Rabbits

Table 2 presents the impacts of dietary Fe levels on the growth performance of growing Rex rabbits. Rabbits in the Fe20, Fe40, and Fe80 groups exhibited a significantly higher ADFI compared with the Fe0 and Fe160 groups (*p* < 0.05), and also showed a significantly higher final BW than that in the Fe160 group (*p* < 0.05). The ADG was markedly higher in the Fe40 group than in the Fe0 and Fe160 groups (*p* < 0.05), and in the Fe20 and Fe80 groups than in the Fe160 group (*p* < 0.05). There was no significant difference in the FCR among the five groups (*p* > 0.05).

### 3.2. Effects of Dietary Fe Levels on Organ Coefficients of Growing Rex Rabbits

As shown in Table 3, among the five groups no significant differences were observed in spleen, liver, and kidney indices (*p* > 0.05).

### 3.3. Effects of Dietary Fe Levels on Meat Quality of Growing Rex Rabbits

Table 4 illustrates the impacts of dietary Fe levels on meat quality parameters. The L* value was observably increased in the Fe20 group compared with the Fe0, Fe80, and Fe160 groups (*p* < 0.05), and was also significantly higher in the Fe40 group than in the Fe160 group (*p* < 0.05). Additionally, the Fe80 group demonstrated a significantly higher a* value than the other four groups (*p* < 0.05).

### 3.4. Effects of Dietary Fe Levels on Fur Quality of Growing Rex Rabbits

Table 5 presents the effects of dietary Fe levels on fur quality parameters. Significantly higher fur areas were observed in the Fe40 and Fe80 groups than in the Fe0, Fe20, and Fe160 groups (*p* < 0.05). There were no significant differences in the fur thickness and hair length among the five groups (*p* > 0.05).

### 3.5. Effects of Dietary Fe Levels on Apparent Nutrient Digestibility of Growing Rex Rabbits

The effects of dietary Fe levels on the apparent nutrient digestibility of growing Rex rabbits are shown in Table 6. Dietary Fe addition markedly increased the apparent digestibility of CP and nitrogen compared to that in the Fe0 group (*p* < 0.05), and the Fe40 rabbits showed a significantly higher apparent digestibility of CP and nitrogen than the Fe20 rabbits (*p* < 0.05). Rabbits in the Fe160 group exhibited a significantly lower apparent digestibility of crude fat than the other four groups (*p* < 0.05). Furthermore, the apparent digestibility of Fe was markedly higher in the Fe20, Fe40, and Fe80 groups than in the Fe160 group (*p* < 0.05).

### 3.6. Effects of Dietary Fe Levels on Serum Biochemical Parameters of Growing Rex Rabbits

As illustrated in Table 7, the serum glucose concentration in the Fe160 group was the highest among the five groups and was significantly higher than that in the Fe0, Fe20, and Fe40 groups (*p* < 0.05). Additionally, rabbits in the Fe40 and Fe80 groups had significantly increased serum glucose levels compared to those in the Fe0 and Fe20 groups (*p* < 0.05). The other parameters among the five groups did not differ significantly (*p* > 0.05).

### 3.7. Effects of Dietary Fe Levels on Serum Immunoglobulin Concentrations of Growing Rex Rabbits

As shown in Table 8, no significant differences were found among the five groups in the serum concentrations of IgA, IgM, and IgG (*p* > 0.05).

### 3.8. Effects of Dietary Fe Levels on Serum Trace Element Levels of Growing Rex Rabbits

Table 9 presents the serum concentrations of Fe, copper, zinc, and manganese in growing Rex rabbits. Dietary Fe supplementation significantly increased serum Fe and copper concentrations relative to those in the Fe0 group (*p* < 0.05). Additionally, serum zinc concentrations were markedly higher in the Fe20, Fe80, and Fe160 groups than in the Fe0 group (*p* < 0.05). No significant difference was found in serum manganese levels among the five groups (*p* > 0.05).

### 3.9. Effects of Dietary Fe Levels on Serum Iron Metabolism Indicator Levels of Growing Rex Rabbits

The levels of Fe metabolism indicators, including HB, Fn, Tf, and TIBC, in the serum are shown in Table 10. The serum HB content was significantly higher in the Fe20, Fe80, and Fe160 groups than in the Fe0 group (*p* < 0.05). In addition, dietary Fe supplementation significantly decreased serum TIBC levels compared with the control group (*p* < 0.05), but the TIBC level in serum in the Fe80 group was significantly increased relative to that in the Fe20, Fe40, and Fe160 groups (*p* < 0.05).

## 4. Discussion

Fe, an essential trace mineral in animals, plays an important role in the maintenance of growth, metabolism, and reproduction [1,22]. In the present study, dietary supplementation with 40 mg/kg Fe increased both the ADG and ADFI in growing Rex rabbits. Liu et al. [7] also reported that supplementing diets with the optimal level of Fe (100 mg/kg) could increase the ADF and FCR in New Zealand meat rabbits. Comparable results have been observed in studies conducted on pigs [23], chickens [24], and fish [25]. Interestingly, higher Fe (80 and 160 mg/kg) supplementation did not further improve the ADG of rabbits. This observation is in line with that of Nassrin et al. [26], who figured out that Fe supplementation above the recommended level (75 mg/kg) had no added value for growing New Zealand white rabbits. Liu et al. [7] also reported that with increasing dietary Fe addition levels from 50 to 200 mg/kg, the ADG and FCR for 60-to-90-day-old Zealand meat rabbits initially increased and then decreased. Previous studies on broilers have shown that high Fe (800 mg/kg) supplementation could decrease growth performance, possibly because excessive Fe increases the metabolic burden on the body and causes oxidative stress [27]. Lee et al. [28] demonstrated that further supplementation of Fe beyond the optimal level led to a linear decrease in the ADG as the dietary Fe level increased in the weaned piglets. Notably, dietary Fe supplementation did not influence the organ indices of the Rex rabbits in this study, which is in keeping with the results in Lin et al. [8]. Collectively, these findings suggest that adding 40 mg/kg Fe to the diet could improve the growth performance without adversely affecting organ development in Rex rabbits.

Nutrient digestibility is a crucial factor that influences animal growth performance. Fe is an important trace element in many organisms and serves as a cofactor for many enzymes such as succinate dehydrogenase and aconitase (ACO), which are involved in the tricarboxylic acid (TCA) cycle, as well as ribonucleotide reductase, amino acid oxidase, and stearoyl CoA desaturase 1 (SCD1), which are involved in biosynthesis pathways [29]. Dietary Fe supplementation has been reported to enhance intestinal digestive enzymes and improve growth performance in juvenile Jian carp [30]. In this study, 20 and 40 mg/kg dietary Fe supplementation increased the digestibility of CP and nitrogen compared to the control group. This finding is consistent with that of Deng et al. [23], who indicated that the dietary addition of 450–600 mg/kg Fe in growing–finishing pig diets elevated the apparent ileal digestibility of amino acids. However, it is important to note that both Fe deficiency and excess negatively affect nutrient digestibility. Fe deficiency may lead to reduced gastric acid secretion, potentially impairing nutrient digestibility by affecting the activity of enzymes like pepsin [31]. Conversely, excess Fe may cause toxicity and cell death through lipid peroxidation and free radical formation, resulting in oxidative stress and impairing digestive system functions [27,32]. Lee et al. [28] reported that higher supplementation of Fe in the diet increased the risk of diarrhea. This could explain the decreased digestibility of crude fat observed in the Fe160 group in the current study. However, the Fe160 group showed a significantly lower Fe digestibility compared with the Fe20, Fe40, and Fe80 groups. The reason might be that once the body’s tissue mineral stores were fully saturated, any excess dietary minerals beyond the required amount were excreted [33]. To sum up, 20 and 40 mg/kg dietary Fe supplementation increased the digestibility of CP and nitrogen in this study.

The Rex rabbit is a domestic rabbit valued for both fur and meat production. In this study, dietary supplementation of 80 mg/kg Fe elevated the a* value of the *longissimus dorsi* muscle 30 min postmortem. Meat color is a crucial visual cue for assessing freshness and quality, and significantly influences consumer purchasing behavior. Studies have demonstrated that the a* value of meat is associated with changes in the chemical properties of myoglobin [34]. Myoglobin is notably prone to oxidation, leading to the conversion of red oxymyoglobin to metmyoglobin and a subsequent decrease in the a* value [35]. Fe, a key component of myoglobin and HB, underlines meat color [8], and plays a critical role in redox processes as a cofactor for several enzymes [36]. Fe-dependent enzymes such as superoxide dismutase (SOD) are crucial for neutralizing free radicals and reducing oxidative stress [37]. SOD converts superoxide anion radicals (O₂⁻) into hydrogen peroxide (H₂O₂), thereby alleviating oxidative damage [38]. However, Fe deficiency and overload can result in oxidative stress [39]. A previous study on broilers also found that, relative to the 50 and 70 mg/kg Fe groups, the 150 mg/kg Fe group elevated the a* value of breast muscle 24 h postmortem [28]. In contrast, in this study the dietary addition of 20 mg/kg Fe increased the L* value of the *longissimus dorsi* muscle 30 min postmortem. Increased L* values are often linked to a higher water content in meat, resulting in a lighter appearance [40], suggesting that low-level Fe supplementation may influence meat water-holding capacity [41]. Seo et al. [42] demonstrated that 100 and 200 mg/kg ferrous methionine dietary addition increased the a* value, and decreased the L* value of the leg and breast muscles. Based on these findings, we propose that dietary supplementation with 80 mg/kg Fe can improve the meat quality of Rex rabbits. In addition, in the present trial the dietary addition of 40 and 80 mg/kg Fe increased the fur area of Rex rabbits. The Fe status of the body is closely related to hair growth and loss [41]. As a key component of HB, Fe is responsible for transporting oxygen to various tissues, including hair follicles, ensuring a sufficient oxygen supply for normal growth and metabolism [43]. Indeed, we observed increased serum HB concentrations in rabbits fed Fe-supplemented diets. Moreover, Fe serves as a cofactor for multiple enzymes participating in cellular functions, particularly cell division and regeneration, thereby promoting hair growth [44]. These findings suggest that supplementation with 40 and 80 mg/kg Fe can improve the fur quality of Rex rabbits, which is particularly important for their value in fur production.

Serum biochemical indicators are the most direct markers reflecting the health status of the body. In this study, rabbits in the Fe160 group had the highest serum GLU concentration. This discovery aligns with that of Choi et al. [45], who indicated that a high dietary Fe intake increased the blood GLU concentration by regulating the expression of glucose-metabolizing enzymes and promoting gluconeogenesis. Moreover, Fe overload can result in insulin resistance and pancreatic β-cells disruption, leading to an increased serum GLU concentration [46]. Dietary Fe supplementation increased serum Fe, copper, and zinc levels, demonstrating a synergistic effect among these three elements in this study. However, no significant differences were found in Fe, copper, and zinc levels in the serum among rabbits fed diets with varying levels of Fe supplementation, which was in keeping with the findings of Liu et al. [7]. The TIBC index is currently used to assess Fe status, and is usually inversely correlated with Fe stores [47,48]. In this study, decreased serum TIBC concentrations were observed in rabbits fed diets supplemented with Fe, which was consistent with the increased serum Fe concentration. A study on pigs also showed that dietary Fe addition linearly increased blood Fe concentrations [33]. These results indicated that the dietary addition of 20-to-160 mg/kg Fe increased Fe stores in Rex rabbits.

## 5. Conclusions

This study demonstrated the multiple benefits of supplementing Rex rabbit diets with appropriate levels of Fe. Dietary Fe supplementation promotes growth performance, enhances the digestibility of crude protein and nitrogen, and improves meat and fur quality in Rex rabbits. Furthermore, it positively influences the serum trace element status of these animals. Specifically, our findings indicate that dietary supplementation with 40 mg/kg Fe (total dietary Fe content of 49.1 mg/kg) significantly improves growth performance, nutrient digestibility, and fur quality, and adding higher doses of iron to the feed will reduce its positive effects in Rex rabbits. However, for optimal meat quality improvement a higher Fe supplementation of 80 mg/kg (total dietary Fe content of 85.6 mg/kg) is recommended. These results provide valuable insights for Rex rabbit farmers and feed manufacturers, potentially leading to improved production efficiency and product quality. Future research should focus on the long-term effects of Fe supplementation and its interactions with other nutrients in Rex rabbit diets.

## Figures and Tables

**Table 1:** Ingredient composition and nutrient levels of basal diets (on an as-fed basis).

Ingredients	Content (%)	Nutrient Concentrations ^2^	Content (%)
Corn	10.00	Digestive energy (MJ/kg)	10.80
Soybean meal	15.00	Crude protein	17.70
Wheat bran	15.00	Crude fat	3.53
Germ meal	15.00	Crude fiber	15.70
Sunflower meal	5.00	Neutral detergent fiber	31.7
Peanut seedling	20.00	Acid detergent fiber	17.10
Alfalfa	10.00	Ash	3.60
Soybean straw powder	5.50	Lignin	4.12
Soybean oil	0.50	Ca	1.20
Mineral and vitamin premix ^1^	4.00	P	0.61

^1^ Premix provided per kg diet: lysine, 7000 mg; methionine, 5500 mg; CaHPO4, 4500 mg; phytase, 1800 IU; choline chloride, 100 mg; Cu, 10 mg; Zn, 60 mg; Mn, 10 mg; I, 1 mg; vitamin A, 8250 IU; vitamin B12, 0.014 mg; vitamin B1, 2.91 mg; vitamin E, 11.85 IU; vitamin B2, 7.2 mg; vitamin D3, 4050 IU; vitamin K3, 1.29 mg; vitamin B6, 2.85 mg; folic acid, 0.72 mg; nicotinamide, 29.4 mg; calcium pantothenate, 21.6 mg; biotin, 0.15 mg; antioxidant, 4.5 mg; bacillus subtilis, 153 trillion CFU; enterococcus faecalis, 3 trillion CFU; bacillus licheniformis, 15 trillion CFU; xylanase, 3600 IU. ^2^ The energy level was calculated and the remaining nutrient levels were measured.

**Table 2:** Effects of dietary Fe levels on growth performance of growing Rex rabbits ^1^.

Items	Dietary Fe Level (mg/kg)					RMSE	*p*-Value
	0	20	40	80	160		
Final BW (g)	2515 ^ab^	2587 ^a^	2569 ^a^	2548 ^a^	2423 ^b^	180	0.006
ADG (g)	15.4 ^bc^	17.8 ^ab^	18.7 ^a^	17.1 ^ab^	13.9 ^c^	5.10	0.004
ADFI (g)	113 ^b^	128 ^a^	131 ^a^	131 ^a^	116 ^b^	5.74	<0.001
FCR (g/g)	7.36	7.26	6.98	7.65	8.35	2.81	0.072

^1^ Results are presented as means with the root mean square error (RMSE). Letters that differ in the same row signify significant differences (*p* < 0.05). *n* = 40.

**Table 3:** Effects of dietary Fe levels on organ indices of growing Rex rabbits ^1^.

Items	Dietary Fe Level (mg/kg)					RMSE	*p*-Value
	0	20	40	80	160		
Spleen index (g/kg)	0.37	0.44	0.49	0.39	0.38	0.13	0.306
Liver index (g/kg)	26.38	23.86	26.08	24.66	25.61	3.03	0.447
Kidney index (g/kg)	4.72	5.08	5.10	4.99	5.02	0.59	0.715

^1^ Results are presented as means with the root mean square error (RMSE). *n* = 40.

**Table 4:** Effects of dietary Fe levels on meat quality of growing Rex rabbits ^1^.

Items	Dietary Fe Level (mg/kg)					RMSE	*p*-Value
	0	20	40	80	160		
pH	6.18	6.28	6.21	6.22	6.33	0.21	0.606
Drip loss	1.639	0.453	0.430	0.423	0.44	1.18	0.099
L*	34.95 ^bc^	38.67 ^a^	36.51 ^ab^	35.16 ^bc^	35.16 ^c^	2.88	0.004
a*	22.92 ^b^	22.92 ^b^	23.46 ^b^	25.07 ^a^	22.22 ^b^	1.48	0.007
b*	−1.75	−2.28	−1.12	−1.80	−1.56	1.07	0.316

^1^ Results are presented as means with the root mean square error (RMSE). Letters that differ in the same row signify significant differences (*p* < 0.05). *n* = 40.

**Table 5:** Effects of dietary Fe levels on fur quality of growing Rex rabbits ^1^.

Items	Dietary Fe Level (mg/kg)					RMSE	*p*-Value
	0	20	40	80	160		
Fur area	1406.22 ^b^	1438.69 ^b^	1536.75 ^a^	1539.62 ^a^	1499.25 ^ab^	87.79	0.013
Fur thickness	2.12	2.37	2.23	2.21	2.61	0.05	0.358
Hair length	2.10	1.96	2.06	2.18	2.00	0.24	0.440

^1^ Results are presented as means with the root mean square error (RMSE). Letters that differ in the same row signify significant differences (*p* < 0.05). *n* = 40.

**Table 6:** Effects of dietary Fe levels on apparent nutrient digestibility of growing Rex rabbits ^1^.

Items (%)	Dietary Fe Level (mg/kg)					RMSE	*p*-Value
	0	20	40	80	160		
Dry matter	62.24	63.59	56.53	58.69	60.10	0.091	0.566
Crude protein	62.26 ^c^	68.45 ^b^	73.24 ^a^	69.36 ^ab^	69.25 ^ab^	3.810	<0.001
Crude fat	83.49 ^a^	87.00 ^a^	83.42 ^a^	81.03 ^a^	72.42 ^b^	0.072	0.004
Crude fiber	21.19	19.51	23.23	20.83	22.56	2.050	0.053
Ash	27.84	31.33	30.00	32.45	24.08	0.104	0.579
Nitrogen	62.26 ^c^	68.45 ^b^	73.24 ^a^	69.36 ^ab^	69.25 ^ab^	3.81	<0.001
Nitrogen utilization	47.21	51.12	53.67	53.43	49.21	7.31	0.387
Biological value of nitrogen	68.83	67.21	72.81	72.68	67.94	9.37	0.682
Ca	59.04	56.51	58.59	51.89	56.96	0.105	0.679
Iron	27.52 ^ab^	29.68 ^a^	31.02 ^a^	29.53 ^a^	27.54 ^b^	4.510	0.028
Zinc	18.69	20.77	18.70	12.90	17.80	0.150	0.531
Manganese	13.45	14.55	10.97	12.67	16.10	0.162	0.979

^1^ Results are presented as means with the root mean square error (RMSE). Letters that differ in the same row signify significant differences (*p* < 0.05). *n* = 40.

**Table 7:** Effects of dietary Fe levels on serum biochemical parameters of growing Rex rabbits ^1^.

Items ^2^	Dietary Fe Level (mg/kg)					RMSE	*p*-Value
	0	20	40	80	160		
ALT (U/L)	60.25	59.87	66.00	60.50	60.87	16.45	0.942
ALP (U/L)	180.37	157.75	154.37	167.37	179.37	45.68	0.701
AST (U/L)	35.75	35.12	38.00	27.87	29.50	13.02	0.482
SDH (IU/L)	432.16	446.79	484.33	480.03	443.53	59.56	0.358
TP (g/L)	65.76	70.41	67.63	66.43	70.47	4.95	0.200
Albumin (g/L)	34.73	34.85	34.77	35.15	36.41	3.46	0.854
Urea (mmol/L)	7.95	8.31	8.04	8.53	8.70	1.02	0.554
Glucose (mmol/L)	5.59 ^c^	5.55 ^c^	5.73 ^b^	5.91 ^ab^	6.80 ^a^	0.65	0.003
Ga (mmol/L)	4.77	4.95	5.04	5.01	5.07	0.25	0.146
P (mmol/L)	2.56	3.06	2.81	2.72	2.95	0.44	0.200

^1^ Results are presented as means with the root mean square error (RMSE). Letters that differ in the same row signify significant differences (*p* < 0.05). *n* = 40. ^2^ ALT, alanine aminotransferase; ALP, alkaline phosphatase; AST, aspartate aminotransferase; TP, total protein; SDH, succinate dehydrogenase.

**Table 8:** Effects of dietary Fe levels on serum immunoglobulin concentrations of growing Rex rabbits ^1^.

Items ^2^ (µg/mL)	Dietary Fe Level (mg/kg)					RMSE	*p*-Value
	0	20	40	80	160		
IgA	138.06	127.15	140.28	137.76	136.04	19.75	0.724
IgM	49.75	58.35	56.03	54.44	53.06	8.07	0.306
IgG	1364.50	1629.90	1727.60	1518.30	1425.60	277.04	0.102

^1^ Results are presented as means with the root mean square error (RMSE). *n* = 40. ^2^ IgA, immunoglobulin A; IgM, immunoglobulin M; IgG, immunoglobulin G.

**Table 9:** Effects of dietary Fe levels on serum trace elements of growing Rex rabbits ^1^.

Items (μmol/L)	Dietary Fe Level (mg/kg)					RMSE	*p*-Value
	0	20	40	80	160		
Iron	31.19 ^b^	40.27 ^a^	40.46 ^a^	38.72 ^a^	36.02 ^a^	4.66	0.005
Copper	27.42 ^b^	31.18 ^a^	30.48 ^a^	30.25 ^a^	30.19 ^a^	2.32	0.027
Zinc	17.41 ^b^	18.92 ^a^	18.08 ^ab^	18.42 ^a^	18.50 ^a^	0.89	0.025
Manganese	3.72	3.64	4.01	3.74	4.08	0.75	0.737

^1^ Results are presented as means with the root mean square error (RMSE). Letters that differ in the same row signify significant differences (*p* < 0.05). *n* = 40.

**Table 10:** Effects of dietary Fe levels on serum iron metabolism indicators of growing Rex rabbits ^1^.

Items	Dietary Fe Level (mg/kg)					RMSE	*p*-Value
	0	20	40	80	160		
Hemoglobin (g/L)	102.28 ^b^	121.02 ^a^	123.94 ^a^	112.60 ^ab^	118.58 ^a^	10.34	0.002
Ferritin (μg/L)	39.01	47.30	45.33	46.71	42.10	6.67	0.123
Transferrin (mg/L)	18.52	19.57	18.54	18.03	19.38	2.92	0.835
Total iron-binding capacity (μmol/L)	61.00 ^a^	51.58 ^c^	52.05 ^c^	57.14 ^b^	52.06 ^c^	3.16	<0.001

^1^ Results are presented as means with the root mean square error (RMSE). Letters that differ in the same row signify significant differences (*p* < 0.05). *n* = 40.

## Data Availability

The original contributions presented in this study are included in the article. Further inquiries can be directed to the corresponding author(s).
